# The simulation of terrible triad injuries in fresh-frozen human cadaveric specimens with intact soft tissue envelope

**DOI:** 10.1007/s00402-022-04677-x

**Published:** 2022-12-13

**Authors:** Fabian Lanzerath, Jutta Knifka, Tim Leschinger, Nadine Ott, Stephanie Kahmann, Michael Hackl, Lars P. Müller, Kilian Wegmann

**Affiliations:** 1 Department of Orthopedic and Trauma Surgery, University Hospital Cologne, Kerpener Street 62, 50937 Cologne, Germany; 2 Department of Anatomy I, Medical Faculty, University of Cologne, Cologne, Germany; 3 OCM (Orthopädische Chirurgie München) Clinic, Munich, Germany

**Keywords:** Fracture simulation, Terrible triad injury, Elbow, Surgical education, Fresh-frozen cadaveric specimens, Intact soft tissue

## Abstract

**Introduction:**

The aim of the present study was to develop a technical process to reproducibly generate terrible triad injuries (TTI) in fresh-frozen human cadaveric specimens, while leaving the skin intact. Such “pre-fractured” specimens, used for scientific analysis and for surgical education, might help to improve current treatment, which is complex and prone to complications.

**Materials and methods:**

To induce the desired fractures, a custom-made fracturing unit was used to apply an axial force on the extended cadaveric elbow specimens, with the forearm pronated and under valgus load. To simulate the valgus load, a pneumatic cylinder was developed to apply valgus stress to the joint by an additional force vector from the lateral side of the joint.

**Results:**

The success rate of TTI induction was 92.3% (12/13). Of the 12 radial head fractures, 3 (25%) were classified Mason type II and 9 (75%) Mason type III. The coronoid fractures were grouped in tip subtype 2 (5 fractures, 41.7%), anteromedial facet (AMF) subtype 2 (4 fractures, 33.3%), AMF subtype 3 (1 fracture, 8.3%) and basal subtype 1 (2 fractures, 16.7%).

**Conclusions:**

The present study provides an instrument for successful and reproducible production of dislocation fracture patterns with their typical accompanying soft tissue lesions. The methodology might be applied on a broad basis to be able to perform biomechanical studies regarding primary stability of fixation concepts for TTI and to educate surgeons in a fairly realistic scenario with the surgical treatment of TTI.

## Introduction

Teaching of surgeons is primarily performed in the clinical setting. Increasing public scrutiny, regulations on working hours, high economic pressure and the steadily rising number of surgical techniques call for new, more efficient training opportunities [1]. Virtual reality simulations, synthetic bone preparations and formalin-fixed or fresh-frozen human cadaveric specimens provide opportunities for surgical training [1]. Dislocation fractures of the elbow are highly demanding to the surgeon and are frequently associated with complications such as stiffness, recurrent joint instabilities, neuropathies of the ulnar nerve, and implant-associated issues [2, 3]. Improved surgical training beyond the operating room might be of help to counteract this. Prerequisite is the availability of corresponding fracture morphologies in cadaveric specimens with an intact skin envelope. Such “pre-fractured” fresh-frozen cadaveric specimens with interindividual anatomy variability would allow a realistic training situation, as recently published by our study group [4]. The biomechanical induction of dislocation fracture patterns is the subject of this work, focusing on terrible triad injuries (TTI).

TTI were first described by Hotchkiss and denote dislocations of the elbow joint in the sense of osteoligamentous injuries [5]. They are composed of the triad of a posterior elbow joint dislocation, a radial head fracture and a fracture of the coronoid process.

An in vivo video analysis of the accident mechanism by Schreiber et al. suggests the elbow joint to commonly dislocate in extension with the forearm pronated, resulting in an axial compression and an initial valgus moment, caused by the inward rotation of the body [6]. In accordance with this, an MRI study by Rhyou et al. suggests a medial injury initiation in posterolateral dislocations of the elbow joint [7]. Fitzpatrick et al. demonstrated that the forearm rotation (pronation) is the primary determinant of the resulting dislocation fracture pattern (TTI) during low-energy axial fracturing without intact soft tissue envelope in a biomechanical study [8]. Fern et al. highlighted the biomechanical effects on joint stability of TTI. [9]

The hypothesis of the study is that it is possible to reproducibly generate TTI based on objective parameters.

## Materials and methods

### Specimens

For the present study, 13 fresh-frozen human cadaveric upper extremities (5 right and 8 left) were available. The specimens were obtained from body donors; written consent was available. Institutional ethics committee approval was given prior to this study (VT 20–1399). The mean age at the time of death was 80 ± 9 (66–94) years. Six of the 13 specimens (46.2%) originated from female donors.

To exclude possible confounding variables for fracture simulation, the specimens were radiographically checked for degenerative changes and implants. In addition, the range of motion was examined under fluoroscopic control, especially to diagnose possible extension and forearm rotation deficits.

CT imaging of each specimen was performed (Phillips iCT 256, Phillips, Amsterdam, Netherlands) and our Institutions Picture Archiving and Communication System (IMPAX) was available to calculate Hounsfield Units (HU) as a reference for bone mineral density in 8 specimens prior to fracturing. Based on the study of Wagner et al. measurements were performed on 3 sequential coronal computed tomography scan slices, however, at the level of the proximal radial–ulnar joint [10]. The region of interest was defined via a circle to best fit the radial head without involving the cortical surface. The mean of 3 measurements was calculated for each specimen.

### Preparation

#### Humerus

After thawing at room temperature, the cadaveric arms were freed from their soft tissue envelope at the mid-humerus and then osteotomized by an oscillating bone saw. The guiding principle for the exact osteotomy localization was that the remaining joints of the upper extremity (shoulder and wrist) could still be used for further scientific purposes. After osteotomy of the humerus, a soft tissue dissection was performed over a length of 5 cm distal to the release point. A raspatory was used to remove remaining tissue from the humerus. Afterwards, the remaining soft tissue was protected by a skin suture.

#### Forearm

**Fig. 1:**
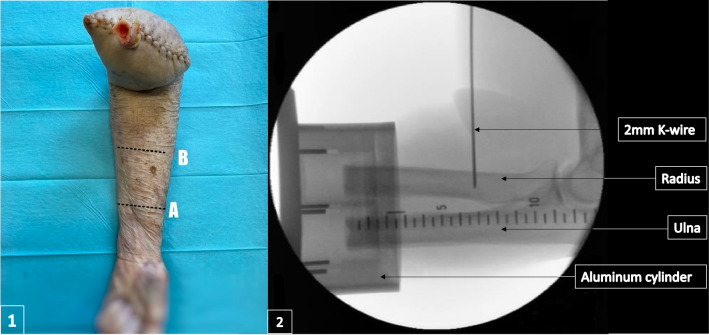
1, **A** marks the planned location of osteotomy, **B** marks the planned drilling location of the *K* wire 7 cm proximal to (**A)**. The specimens’ arm was supinated for this photograph. However, the *K* wire drilling took place in 60° pronation; 2, Radiological verification of the correct *K* wire position in the radius

The specimens’ arm was flexed 90° at the elbow joint and the forearm was rotated into 60° of pronation by use of a standard goniometer. In this position, a 2 mm *K* wire was drilled parallel to the longitudinal axis of the humerus, 7 cm proximal to the planned site of forearm osteotomy (Fig. 1). This method was developed to ensure the correct rotation of the forearm (60°), even after the subsequent osteotomy: If the elbow joint was now 90° flexed and the *K* wire aligned parallel to the humerus, the forearm was 60° pronated. The correct position of the *K* wire was verified under radiographic control. Now, the forearm osteotomy was be performed. The osteotomy level was 7 cm distal to the drilling site of the *K* wire. The forearm was subsequently freed from its soft tissue envelope over a length of 5 cm proximal to the release point with the assistance of a raspatory. Only the interosseous membrane of the forearm was left intact to maintain its stabilizing influence. The soft tissue of the forearm was also sutured.

#### Specimen fixation

To clamp the cadaveric specimens into the fracturing unit, the humerus as well as radius and ulna were fixed into custom-made aluminum cylinders with a 4 mm-thick foundation. To embed the specimens, we used polymethyl methacrylate (PMMA). During the potting process of both, radius and ulna, into a shared aluminum cylinder, care was taken to ensure that the elbow joint was flexed 90° and the *K* wire remained parallel to the humerus, thus maintaining 60° of pronation. The foundation of the aluminum cylinder was aligned perpendicular to the longitudinal axes of the humerus and radius and ulna, respectively, to allow precise axial force induction.

#### Fracturing unit and fracture simulation

**Fig. 2:**
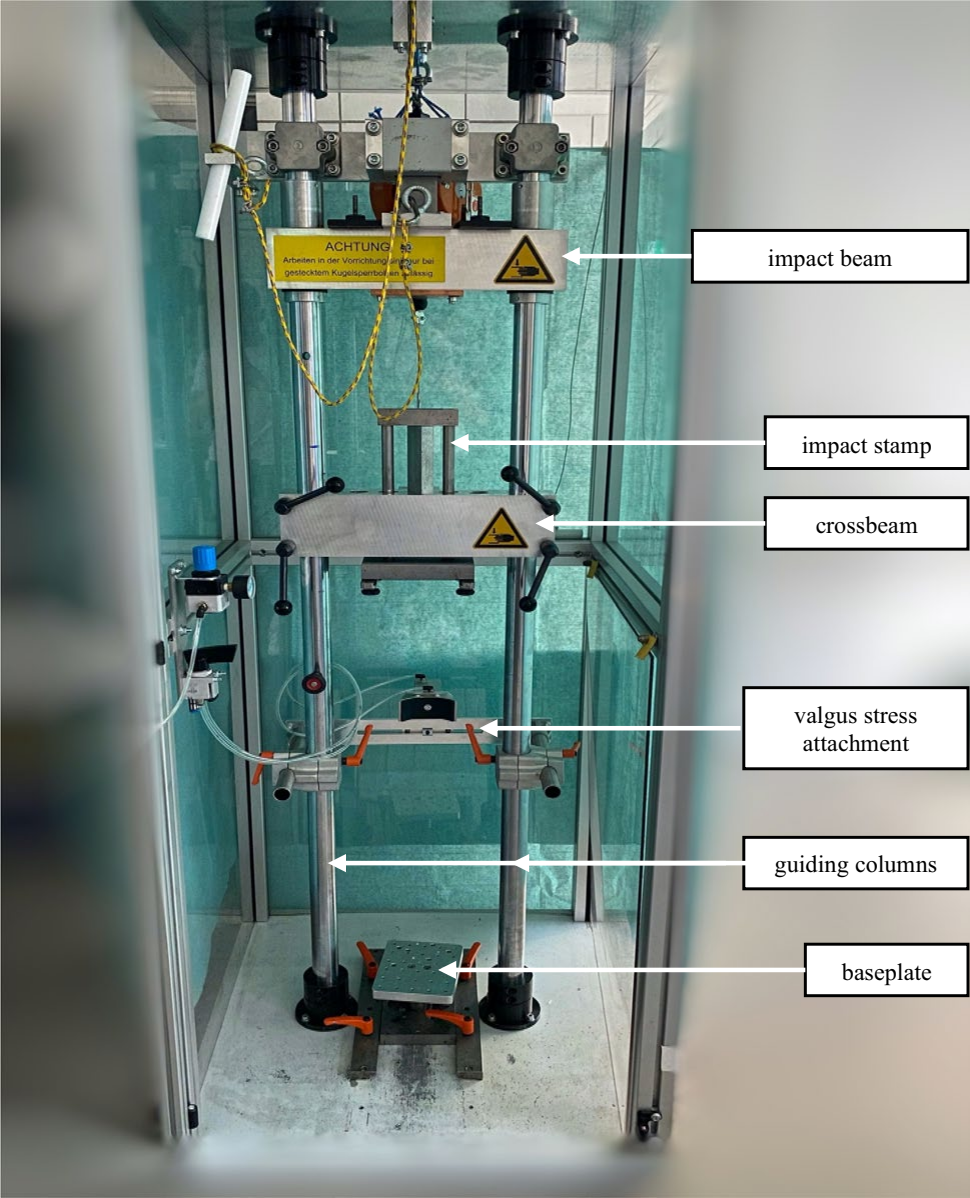
Fracture unit set-up

Fracturing of the cadaveric specimens was performed in a custom-made fracturing unit as used in earlier work by the study group (Fig. 2) [11–13].

The unit mainly consists of two vertical guiding columns, which carry an impact beam and a crossbeam, both height adjustable. The impact beam is guided by the guiding columns in a vertical direction and can act on an impact stamp, which itself is led by the crossbeam. The impact stamp features a contact plate at its upper and lower end. The upper contact plate communicates with the impact beam, the lower contact plate can be connected to the foundation of the proximal aluminum cylinder in which the humerus was previously fixed. The impact stamp exhibits a length of more than 120 mm, by that allowing appropriate impaction. The distal aluminum cylinder comes to rest on a base plate at the bottom of the fracturing unit. As the crossbeam is height-adjustable, specimens of different lengths can be brought into the desired position.

The height from which the impact beam is dropped and the weight acting on the impact stamp can be adjusted individually. Dropping the impact beam onto the upper contact plate of the impact stamp pushes the stamp downwards, resulting in compression of the specimen.

**Fig. 3:**
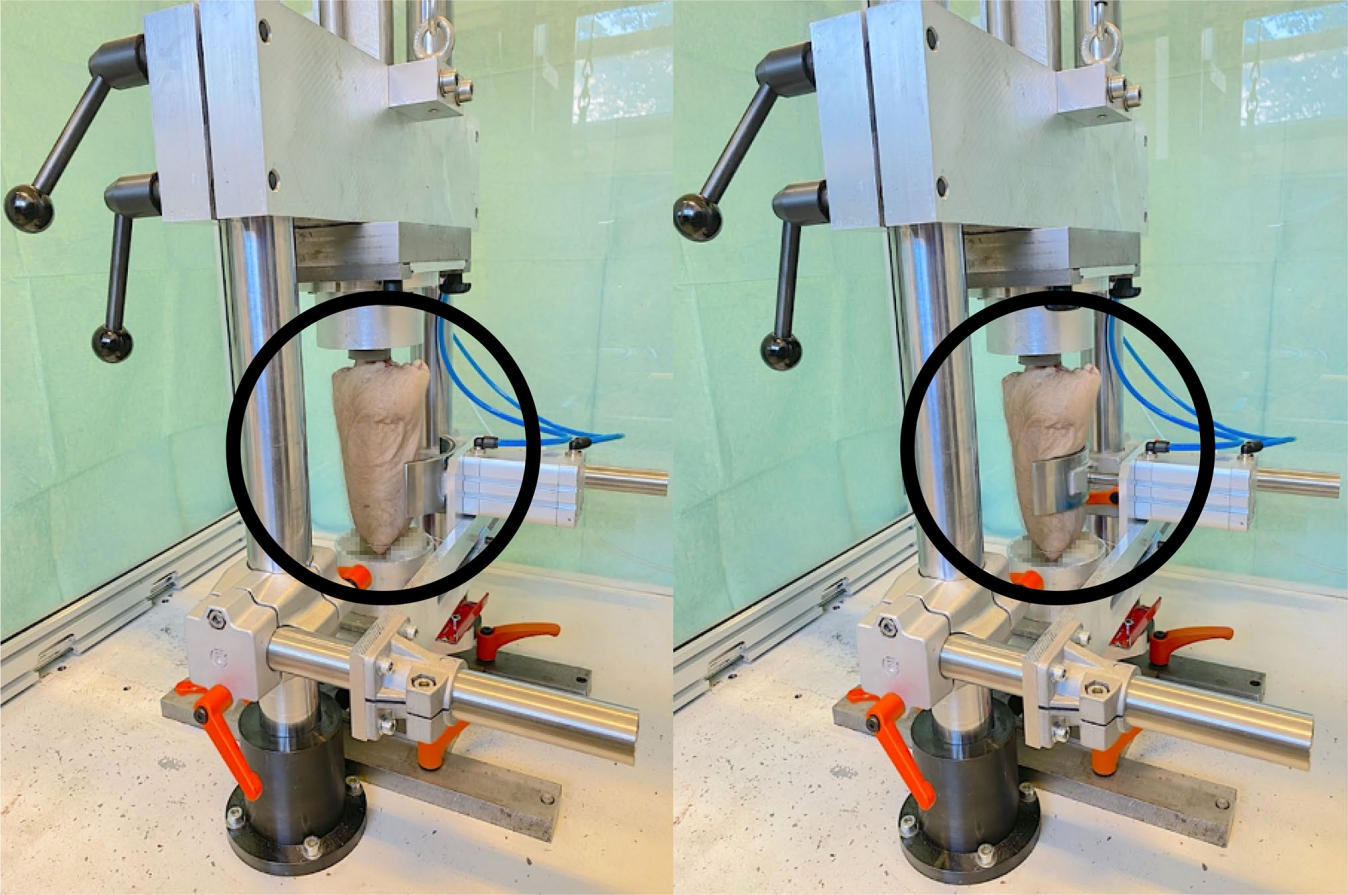
Generation of valgus stress at the specimen’s elbow joint by extending the pneumatic cylinder

Based on empirical data of elbow fracture induction, kinetic energies of 50 J were applied for fracturing. In the event of the necessity to perform a second hit, the applied energy was increased to 75 J. The drop weight was 15 kg in all cases. To apply the desired energy, a height of 34 cm was set for the first hit and a height of 51 cm in case of a required second hit. The energy to be applied was calculated using the formula of potential energy ($\mathrm{E}=\mathrm{m}\times \mathrm{g}\times \mathrm{h}$; E = potential energy, m = mass, g = gravitational acceleration, h = height). To simulate the postulated initial valgus moment, a valgus stress add-on device was designed to be attached to the two vertical guiding columns (Fig. 3). Thus, valgus stress can be generated in addition to axial compression. The power arm is operated pneumatically. The pneumatic cylinder allows a perpendicular force between 6 Newton (N) and 500 N, calculated by the formula: force = piston area × air pressure.

In a first series of simulations, 7 specimens were fractured merely axially in extension and pronation. Subsequently, the remaining 6 specimens were additionally exposed to 50 N valgus stress via the add-on device.

Artificial damping mechanisms during fracturing were not applied and the internal friction of the pneumatic cylinder neglected in the calculation of the acting forces.

The prepared specimens were clamped into the fracturing unit in full extension and the previously set 60° of pronation, with axial application of the impulse and, in the testing group, the additional valgus load.

X-ray and CT imaging were conducted to analyze the emerging injuries. All fractures were classified independently by two trauma surgeons (TL, KW). Radial head fractures were classified according to a modified Mason classification and fractures of the coronoid process according to O´Driscoll [14, 15]. Mason type I fractures were defined as fractures with a fragment dislocation of < 2 mm or involvement of < 30% of the articular surface, type II fractures as fractures with a 2–5 mm dislocation and involvement ≥ 30% of the articular surface and type III fractures as fractures with a fragment dislocation of > 5 mm or multiple fractures of the radial head.

### Statistical analysis

Statistical analysis was performed using IBM SPSS Statistics Version 27 for macOS (IBM, New York, USA). Means, standard deviations, and ranges were calculated. Associations between the number of radial head fracture fragments (single fragment/multi-fragment) and HU were quantified using Spearman’s rank correlation. Pearson’s correlation was used for the association between specimens’ age at the time of death and HU. The level of significance was defined as *p* ≤ 0.05 (two-sided tests).

## Results

The characteristics of the specimens are listed in Table 1. Multi-fragmented radial head fractures were observed in 9 out of 13 cadaveric specimens (69.2%) and single-fragmentary fractures resulted in 4 cadaveric specimens (30.8%). The measured HU average was 277 ± 42 (208–323). The HU average in the radial head was 261 for multi-fragmentary fractures and 304 for single-fragment fractures. Two specimens (no. 6 and no. 10) required a second hit. The amount of impaction of the impact stamp, the so-called braking distance, averaged 1.8 ± 1.0 (0.8–4.6) cm.

**Table 1:** Individual data overview of all specimens

Specimen	Side	Gender	Age, year	HU	Fragments	Height, cm	Weight, kg	Braking distance, cm	Energy, *J*	Valgus force, *N*	TTI	Mason	O’Driscoll
1	R	F	75	N/A	Multi	34	15	2.0	50.0	–	Yes	Type III	Tip subtype 2
2	L	M	83	317	Multi	34	15	1.2	50.0	–	Yes	Type III	Tip subtype 2
3	L	F	86	278	Single	34	15	1.5	50.0	–	Yes	Type II	Tip subtype 2
4	L	F	88	232	Multi	34	15	2.5	50.0	–	Yes	Type III	AMF subtype 2
5	R	F	88	208	Multi	34	15	4.6	50.0	–	Yes	Type III	Basal subtype 1
6	L	M	69	N/A	Multi	51	15	2.2	75.0	–	Yes	Type III	Basal subtype 1
7	R	F	90	N/A	Multi	34	15	1.7	50.0	–	Yes	Type III	AMF subtype 3
8	R	F	77	N/A	Single	34	15	1.5	50.0	50	Yes	Type II	AMF subtype 2
9	R	M	69	N/A	Multi	34	15	1.4	50.0	50	Yes	Type III	Tip subtype 2
10	L	M	66	294	Multi	51	15	1.2	75.0	50	Yes	Type III	AMF subtype 2
11	L	M	83	323	Single	34	15	1.9	50.0	50	Yes	Type II	AMF subtype 2
12	L	M	71	311	Single	34	15	0.8	50.0	50	No	Type II	–
13	L	M	94	252	Multi	34	15	0.9	50.0	50	Yes	Type III	Tip subtype 2

*yr*. years, *HU* hounsfield units, *Height* height from which the impact beam was released, *Weight* weight of the impact beam, *Braking distance* refers to the stamp, *Energy* potential energy, *J* joule, *N* newton, *R* right, *L* left, *F* female, *M* male, *N/A* not available, *TTI* terrible triad, *AMF* anteromedial facet

The fragments (single fragment/multi-fragment) and HU of the radial head correlated negatively, however, not significant (*r* = − 0.507; *p* = 0.2; *n* = 8). Radial head HU and age of the cadaveric specimens showed a negative, insignificant correlation. (*r* = − 0.556; *p* = 0.152, *n* = 8).

**Fig. 4:**
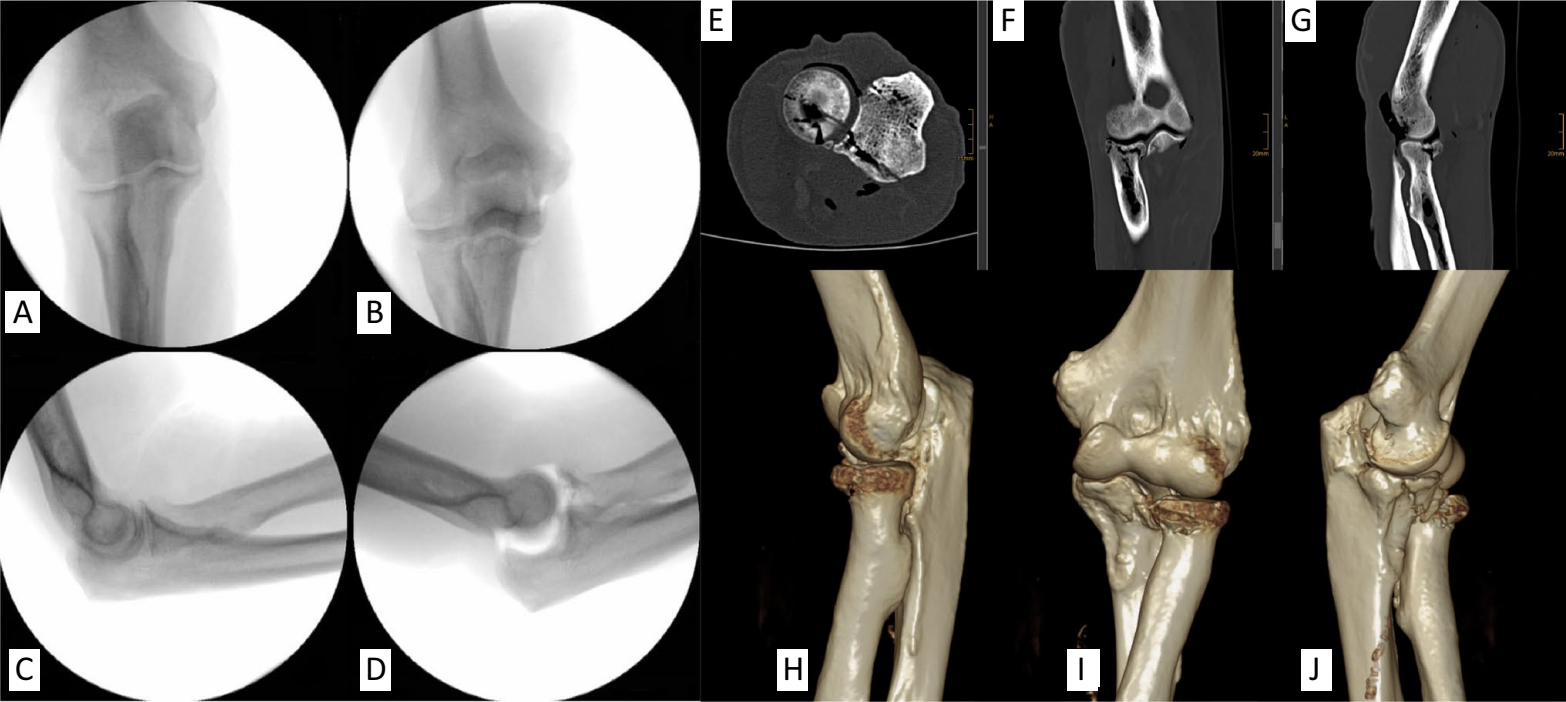
Typical induced fracture pattern exemplified by the diagnostics of a cadaveric specimen (no. 2). X-ray images before (**A** and **C**) and after fracturing (**B** and **D**). CT images, E: axial plane, F: coronal plane, G: sagittal plane. 3D-reconstruction, H: lateral view, I: ventral view, J: medial view, preserved integrity of the sublime tubercle and the AMF with simultaneous fracture of the coronoid’s tip. The fracture line runs in the frontal plane

**Fig. 5:**
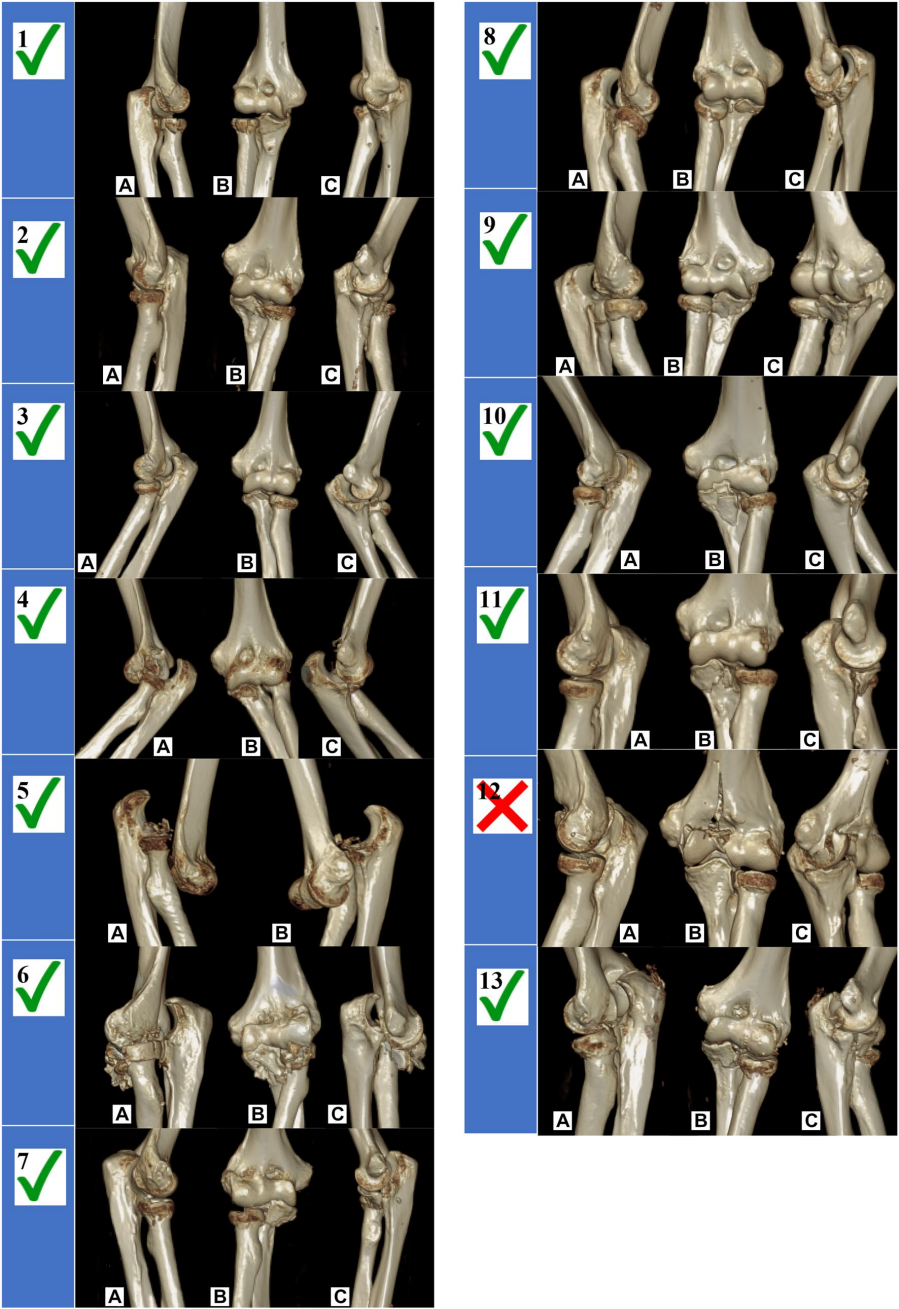
Overview of 3D fracture reconstruction; Left side (1–7) without and right side (8–13) with valgus load

The success rate of TTI induction was 92.3% (12/13). A typical fracture is shown in Figs. 4, 3D reconstructions of all induced fractures are shown in Fig. 5. Regarding the successful simulations, 3 (25%) Mason type II and 9 (75%) Mason type III radial head fractures were induced. Concerning the coronoid, there were 5 (41.7%) tip subtype 2 fractures, 4 (33.3%) AMF subtype 2 fractures, 1 (8.3%) AMF subtype 3 fracture and 2 (16.7%) basal subtype 1 fractures. In one specimen (no. 12), only the radial head was fractured while the coronoid remained intact. Instead of the coronoid, the medial distal humerus was fractured articularly.

## Discussion

In contrast to previous studies on the simulation of bony fractures without a dislocation mechanism and publications on the low-energy production of TTI with extensively dissected soft tissue envelope, the present study represents the first work on production of TTI with an intact skin envelope. The success rate of TTI production was 92.3% (12/13). The hypothesis that objective parameters would allow reproducible generation of complex dislocation fractures based on the simulation of the accident mechanism postulated by Schreiber et al. was confirmed [6].

Interestingly, the only unsuccessful attempt (no. 12) also resulted in joint dislocation with concomitant fracture of the radial head, while the coronoid remained intact. Instead of the latter, the medial distal humerus was fractured with involvement of the articular surface.

The methodology of the study provides the first guidance for simulating TTI to practice surgical operation procedures from skin incision to skin suture under realistic conditions. In particular, the preservation of skin, subcutaneous fat, muscle, and nerves makes individual treatment planning and access to the fracture comparable to the actual intraoperative situation. This is equally true for the risks to be considered in fracture care of TTI (e.g., ulnar nerve lesion due to topographical proximity in medial approaches). Thus, the described experimental set-up might be adopted to allow trainee surgeons, as well as experienced surgeons, to treat realistic dislocation fracture patterns in a low-stress environment without endangering patients. As recently evaluated by our study group, such a course format, in which pre-fractured specimens are surgically treated under the guidance of designated experts, would provide an effective surgical trauma care intervention teaching tool [4]. Moreover, the technical process described can be applied to enhance the understanding of elbow pathobiomechanics and to be able to perform biomechanical studies regarding primary stability of fixation concepts for TTI.

In the literature, Mason type II or III radial head fractures are most commonly described in the context of TTI [16]. In the present work, Mason type II fractures were induced in 3 of the 12 successful TTI (25%). Type III fractures were induced in the remaining 9 cadaveric specimens (75%). Regarding the coronoid, fractures of the tip (subtype 1 and 2) are predominantly reported in TTI clinically [16]. This biomechanical study observed 5 (41.7%) tip subtype 2 fractures, 4 (33.3%) AMF subtype 2 fractures, 1 (8.3%) AMF subtype 3 fracture, and 2 (16.7%) basal subtype 1 fractures.

It is critical to note that although TTI were induced, these typically result from posterolateral rotatory instability, whereby typically only the anterolateral facet of the coronoid is affected in terms of a tip fracture. A classification that respects the fracture morphologies in combined injuries of the radial head and the coronoid process, without simply grouping them together as TTI, is the Writington classification [17]. A common TTI would be classified as a “C” according to this classification. In contrast, a combined injury to the radial head and both facets of the coronoid would be classified as a “B + ” injury. Possibly, even under valgus loading, the test bench does not allow sufficient rotational and dislocation components to prevent distancing and thus shearing of the AMF. This is also shown by the course of the fracture line, which can be traced in Fig. 5: in a clinically typical fracture of the AMF, the fracture line is concave according to the shape of the trochlea. Instead, the fracture lines of the AMF fractures generated in this work tend to extend in a strictly frontal plane into the AMF. The pneumatic cylinder allows a valgus force of up to 500 N. In our test setup, only 50 N were used. It might be necessary to adjust the force to a higher level at this point. However, surgical fracture treatment in practice might benefit from the generated injury patterns, as the involvement of the AMF acquires biomechanical relevance and should be treated accordingly.

The energies applied might be used as a guideline in the future. The cost of the standard HU measurement based on CT scans obtained in advance must be critically evaluated. The initial measurement of HU has not yet been validated for the proximal radioulnar joint. There is currently no correlation with bone density measurements, which are classically assessed by dual x-ray absorptiometry. Wagner et al. correlated HU in the distal radioulnar joint with conventional dual x-ray absorptiometry only [10]. One likely explanation for the fact that the correlation between radial head fragments and HU did not show significance is the small number of available cadaveric specimens. Conventional fluoroscopic scans prior to fracturing were found to be sufficient to exclude possible confounding variables such as degenerative changes, implants, or range of motion deficits.

The mean age of the specimen in this study does not correspond to the incidence peak of the injury (approximately 4th decade of life) [18]. However, this fact is negligible as the specimens are intended for use in surgical training and accordingly do not require representativeness with respect to age, provided the characteristic fracture patterns are present and the individual anatomy is preserved.

## Conclusion

TTI of the elbow were successfully induced by applying a high-energy impulse onto human cadaveric specimens while preserving the soft tissue envelope. The described methodology could serve as an approach to broaden knowledge regarding its pathobiomechanics, to perform biomechanical studies regarding its primary stability of fixation concepts and might allow surgeons to train in a low-stress environment beyond the operating theatre without putting patients at risk.
